# Understanding and Controlling Sialylation in a CHO Fc-Fusion Process

**DOI:** 10.1371/journal.pone.0157111

**Published:** 2016-06-16

**Authors:** Amanda M. Lewis, William D. Croughan, Nelly Aranibar, Alison G. Lee, Bethanne Warrack, Nicholas R. Abu-Absi, Rutva Patel, Barry Drew, Michael C. Borys, Michael D. Reily, Zheng Jian Li

**Affiliations:** 1 Biologics Development, Global Manufacturing and Supply, Bristol-Myers Squibb Company, Devens, MA, United States of America; 2 Research and Development, Bristol-Myers Squibb Company, Princeton, NJ, United States of America; University of Nebraska Medical Center, UNITED STATES

## Abstract

A Chinese hamster ovary (CHO) bioprocess, where the product is a sialylated Fc-fusion protein, was operated at pilot and manufacturing scale and significant variation of sialylation level was observed. In order to more tightly control glycosylation profiles, we sought to identify the cause of variability. Untargeted metabolomics and transcriptomics methods were applied to select samples from the large scale runs. Lower sialylation was correlated with elevated mannose levels, a shift in glucose metabolism, and increased oxidative stress response. Using a 5-L scale model operated with a reduced dissolved oxygen set point, we were able to reproduce the phenotypic profiles observed at manufacturing scale including lower sialylation, higher lactate and lower ammonia levels. Targeted transcriptomics and metabolomics confirmed that reduced oxygen levels resulted in increased mannose levels, a shift towards glycolysis, and increased oxidative stress response similar to the manufacturing scale. Finally, we propose a biological mechanism linking large scale operation and sialylation variation. Oxidative stress results from gas transfer limitations at large scale and the presence of oxygen dead-zones inducing upregulation of glycolysis and mannose biosynthesis, and downregulation of hexosamine biosynthesis and acetyl-CoA formation. The lower flux through the hexosamine pathway and reduced intracellular pools of acetyl-CoA led to reduced formation of N-acetylglucosamine and N-acetylneuraminic acid, both key building blocks of N-glycan structures. This study reports for the first time a link between oxidative stress and mammalian protein sialyation. In this study, process, analytical, metabolomic, and transcriptomic data at manufacturing, pilot, and laboratory scales were taken together to develop a systems level understanding of the process and identify oxygen limitation as the root cause of glycosylation variability.

## Introduction

Glycosylated protein therapeutics are an important class of drugs used to treat a variety of medical indications ranging from cancers, blood disorders, degenerative diseases, autoimmune disorders, and cardiac disease [1]. The vast majority of these proteins are produced using mammalian cellular hosts including mouse, human and Chinese hamster ovary (CHO) cell lines because of their capacity for post-translational modifications including glycosylation. The glycan structure of a protein is known to impact its therapeutic properties, half-life, bioactivity, solubility, and antigenicity [2–4]. One such glycan modification, sialylation, refers to the addition of N-acetylneuraminic acid (NANA) to the glycan terminus. The presence of terminal NANA has been shown to impact many key properties of glycosylated proteins, including protein stability, circulatory half-life, solubility and thermal stability [5–7].

Because glycan structure can impact protein properties, a consistent glycosylation profile is desirable, and demonstration of this consistency is typically required by regulatory agencies such as the FDA [1]. However, it is well known that environmental factors including bioprocessing conditions impact glycosylation structure [1,2,8–10]. This includes, but is not limited to, osmolality, glucose, dissolved oxygen, bioreactor pH, ammonia, glutamate, glutamine, pCO_2_, temperature, and feed composition. While such studies provide a critical foundation for understanding glycosylated therapeutic proteins, process conditions have been shown to have varying impacts depending on cell type, molecule, and even scale [1,2]. Understanding the link between process conditions and glycan structure could enable greater control of processes, and the ability to fine tune glycosylation in a predictable manner. A different set of variables that enable this fine-tuning could be needed for each bioprocess. By coupling traditional bioprocessing experiments with systems biology methods, it may be possible to more rationally identify these control variables, and use them to develop robust processes [11].

In this study, we examine the sialylation profile of a CHO process used to produce an Fc-fusion product where sialic acid, measured as the molar ratio of NANA to protein, is a quality attribute. Significant run to run variability for a given culture duration is observed over pilot (50, 500, and 900-L) and manufacturing (5000-L) scales. This system was further examined using advanced analytical methods in order to gain insights into potential sources of this variation to enable strategies to achieve more consistent product quality profiles. Utilizing metabolomics and transcriptomics techniques, the underlying biological sources of sialylation variation in the process are identified as glucose consumption and oxidative stress. Previous reports regarding the impact of oxygen on mammalian protein glycosylation are limited and conflicting [10,12,13]. Using a laboratory (5-L) scale-down model of the process, the ability to control and tune sialylation levels by manipulating oxygen availability is demonstrated. The primary aim of this study was to understand the causes of sialic acid variation at manufacturing scale, and use that understanding to better control sialylation, ultimately resulting in a more robust process.

## Materials and Methods

### CHO Cell Culture Conditions

A CHO DG44 cell line expressing a recombinant antibody fusion protein using a vector with the dihydrofolate reductase-deficient (DHFR) selection marker was used for all experiments. Seed culture was expanded to an appropriate volume using baffled shake flasks maintained at 185 rpm with a 3/4 inch throw and 5% CO_2_. The experiments all used the same proprietary, chemically defined media. Laboratory scale experiments were carried out in glass vessel 5-L bioreactors with an initial working volume of 3.5 L. Additional data was collected from large scale runs, including 50-L, 500-L, 900-L and 5000-L scale stainless steel bioreactors. The initial working volumes were 30-L, 300-L, 600-L, and 3000-L respectively. The temperature, initially controlled at 37°C, was reduced to a lower temperature at a pre-defined time to extend the viability and productivity of the culture. The pH in the bioreactor was controlled through the addition of CO_2_ gas and 1 M Na_2_CO_3_. The bioreactors were operated in fed-batch mode with daily feed addition based on the initial working volume. Basal and feed media are chemically defined, proprietary formulations. Dextran sulfate was added according to pre-determined criteria. A concentrated bolus of glucose was added to the bioreactors if the concentration of glucose fell below a predetermined threshold.

Culture performance was monitored with daily samples, measuring cell density and viability using a ViCell (Beckman Coulter, Inc., Indianapolis, IN) automated cell counter that operates using the trypan blue dye exclusion method. Monitoring of pH, pCO_2_, and pO_2_ was performed using a pHOX or FLEX (both from Nova Biomedical, Waltham, MA) instrument. Monitoring of glucose, lactate, glutamine, glutamate, galactose, and ammonium was performed using a Roche Cedex HT (Roche Custom Biotech, Mannheim, Germany) or NOVA FLEX instrument. Periodically, culture supernatant and cell pellets were prepared. Culture broth was centrifuged at 1000 rpm for 8 minutes to separate the supernatant from biomass. Supernatant aliquots were frozen directly for metabolomics and analytical testing. Cells were washed twice with cold PBS and frozen in aliquots of 10 x 10^6^ cells (transcriptomics) or 100 x 10^6^ cells (intracellular metabolomics).

### Transcriptomics

Whole cell RNA was extracted from cell pellets using the RiboPure kit (Ambion, a division of Thermo Fisher Scientific). RNA was converted to cDNA using the RT^2^ First Strand Synthesis Kit. Quantitative PCR was carried out using SYBR Green Master Mix and PCR arrays (SABiosciences, a division of Qiagen, Valencia, CA). The Glucose Metabolism (PAJJ-006Z) and Oxidative Stress (PAJJ-065Z) arrays, optimized for Chinese Hamster, were used for these studies. The ViiA 7 Real-Time PCR System (Applied Biosystems) and software was used to run RT-PCR and analyze results. The comparative C_T_ method was used to normalize measurements relative to one of five established housekeeping genes (HKGs) included in the arrays; Actb, Actr5, B2m, Gapdh and Hgprt. In these studies, Hgprt was selected as the HKG for normalization because it exhibited the most consistent gene expression level across all samples tested. After normalizing, relative mRNA expression for each gene of interest was made across conditions using the comparative C_T_ method. A fold change greater than 2 was considered statistically significant.

Whole cell RNA was treated with DNAse 1 and RNase inhibitor enzyme prior to providing select samples to Expression Analysis (Durham, NC) for global microarrays. RNA integrity was checked by electrophoresis using an Agilent Bioanalyzer 2100. All subsequent processing steps were performed by Expression Analysis. Affymetrix CHO Gene ST arrays were used to measure expression. Robust Multi-array Average (RMA) normalization was used for array comparison prior to filtering for statistical significance.

### Metabolomics Sample preparation and NMR data acquisition

Supernatant samples were obtained by centrifugation to remove cells. 0.5 mL of supernatants were mixed with 0.25 mL of 0.2 M phosphate buffer in 20% D_2_O, pH 7.0 (meter reading uncorrected for deuterium isotope effect) containing two reference standards: 0.3 mM 1,1,2,2,3,3-hexadeutero-3-pentane sulfonic acid (DSS-d6) and 0.1 mM of 1,1-difluoro-1-trimethylsilanylmethylphosphonic acid (DFTMP) as an internal pH reference. Dried cell extracts (prepared from about 100 million cells) were stored -80°C and then prepared by dissolving in two parts of D_2_O 99.9% isotopic purity (Cambridge Isotope Laboratories, Tewksbury, MA) and one part of deuterated 0.2 M phosphate buffer (prepared as described above for supernatants) for a total volume of 0.6 mL.

0.6 mL samples of supernatants or cell extracts were transferred to 5 mm NMR tubes and queued up in a sample changer SampleJet (Bruker Analytik, Rheinstetten, Germany) equipped with a cooled storage unit (at 4–6°C). Acquisition of spectra was at 27°C. 1D proton NMR spectra of supernatants and cell extracts were measured on a Bruker 600 MHz NMR spectrometer (Bruker Analytik, Rheinstetten, Germany) equipped with a 5 mm TCI cryoprobe. A constant number of scans (256 for supernatants and 512 for cell extracts) and receiver gain were used to facilitate comparisons of relative analyte concentrations between samples. The pulse sequence used was a 1D version of a NOESY experiment with gradient water suppression during nOe mixing time of 0.05 s and during a relaxation time of 2 s. The 90° pulse width was automatically determined for each sample. All free induction decays (FID’s) were subjected to Fourier transformation and automatic phase and baseline correction prior to calibrating to DSS at 0 ppm. The quantitative analysis was performed by integration of peaks based on the chemical shifts and peak shapes of known spectral standards, utilizing the Multi-integrate routine of AMIX Analysis of MIXtures) software (version 3.9, Bruker Analytik).

### Metabolomics Sample preparation for Liquid Chromatography/Mass Spectrometry

Frozen supernatant samples were thawed at room temperature for two hours and gently vortexed. A 50 μL aliquot of each sample was transferred to the corresponding well of an Axygen 96-well plate (Corning Life Sciences, Edison, NJ). 150 μL of ice-cold methanol containing 0.1% formic acid was added to each sample. The plate was vortexed for 1 min and then spun for 10 min at 5000 rpm in a Beckman Coulter Allegra 25 centrifuge with a TA 10.25 rotor (Beckman Coulter Inc., Indianapolis, IN). 50 μL of supernatant was transferred to a new 96-well plate for reversed phase LC/MS analysis and a second 96-well plate was similarly prepared with another 50 μL aliquot for HILIC LC/MS analysis.

Frozen cell extracts were thawed at room temperature for two hours. Samples were reconstituted with 100 μL of ice-cold methanol with 0.1% formic acid and vortexed for 1 min. 100 μL of water was added to each sample followed by additional vortexing. Tubes were spun for 10 min at 4000 rpm. 100 μL aliquots were transferred to corresponding wells of a 96-well plate for reversed phase LC/MS analysis and a second 96-well plate was similarly prepared for HILIC LC/MS analysis. Plates were dried using a V&P Scientific Model VP-177 96-well plate dryer (V&P Scientific, Dan Diego, CA) with nitrogen gas for approximately 6 hours prior to storage in a -80°C freezer and processing for UHPLC high-resolution LC/MS analysis.

### Reversed Phase LC/MS

The reversed phase plate was reconstituted with 90:10 water:methanol. An internal standard mixture containing d5-glutamic acid, d3-carnitine, d8-phenylalanine, d5-hippuric acid, d16-sebacic acid, d4-palmitic acid, d3-octanoyl carnitine, and d4-deoxycholic acid was added to all samples. This internal standard mixture was used to ascertain that the mass spectrometer performance was stable during the analysis of the set. Samples were analyzed by LC/MS using an Accela UHPLC interfaced to an Exactive Plus ion trap mass spectrometer with a HESI source (ThermoFisher Scientific, San Jose, CA). Chromatographic separations were achieved employing a 2.1 x 150 mm, 1.7μm, Acquity BEH C18 column (Waters, Milford, MA) with gradient elution at 0.6 mL/min. The column temperature was maintained at 65°C. Mobile phase A was water with 0.1% formic acid and mobile phase B was 98:2 acetonitrile:water with 0.1% formic acid. Mobile phase A was held at 100% for 0.5 min and then a three step linear gradient was formed from 0% to 20% mobile phase B over 2.5 min, to 60% mobile phase B in 1 min and then to 100% phase B in 3 min. The final composition was held for 2 min before returning to the initial conditions. Positive and negative electrospray ionization (ESI) data were acquired (separate injections) with a mass accuracy within 5 ppm at 35,000 resolution. A single 10 μL injection was used for each ionization mode. Instrumental settings follow: maximum injection time 10 msec, capillary temperature 320°C; tube lens voltage 175 V; ESI spray voltage 4.3 kV for positive ion mode, 3.6 kV for negative ion mode; sheath gas 2 arbitrary units (arbs).

### HILIC/MS

The HILIC plate was reconstituted with 45:45:10 methanol:acetonitrile:water. The internal standard mixture was added to all samples and they were analyzed by LC/MS as described above. Chromatographic separations were achieved employing a 2.1 x 150 mm, 1.7 μm, Acquity BEH Amide column (Waters, Milford, MA) with gradient elution at 0.3 mL/min. The column temperature was maintained at 65°C. Mobile phase C was 95:5 water:acetonitrile with 10 mM ammonium acetate and 0.05% ammonium hydroxide. Mobile phase D was acetonitrile with 0.05% ammonium hydroxide. A linear gradient was formed from 5% to 63% mobile phase C over 3.5 min. The final composition was held for 3.5 min before returning to the initial conditions.

Peak areas for LC/MS quantification of 246 metabolites were calculated using Component Elucidator, a software package developed at Bristol-Myers Squibb [14].

### Analytical Attributes

Protein titer was quantified by HPLC using an affinity chromatography (protein A) column based on reference standards of the purified product at known concentrations. The sialic acid compound NANA was cleaved from protein by mild acid hydrolysis by adding 2N sulfuric acid to the sample and incubating at 80°C for one hour. The released NANA was then separated and quantified by Waters HPLC alliance 2695 using a Rezex Monosaccharide RHM ion exchange column 8 μm, 7.8 x 300 mm and 8 μm, 7.8 x 50 mm guard column (Phenomenex, Torrance, CA). Elution was carried out isocratically at 40°C with 5 mM sulfuric acid with a flow rate of 0.6 mL/min. The sialic acid was monitored at an absorbance of 206 nm and quantitated via peak area and linear regression of a standard curve using 2487 dual wavelength detector. The results were reported as a molar ratio of NANA to protein.

The chromatographic profiles of N-linked oligosaccharides were achieved by enzymatic hydrolysis with PNGase F for 22 hours. The free oligosaccharides were profiled using high pH anion exchange chromatography employing electrochemical detection on an HPLC system. CarboPac PA-1 4 X 250 mm and CarboPac PA-1 4 X 50 mm (Dionex Corporation, Sunnyvale, CA) were used on a Waters Alliance HPLC system (Milford, MA) equipped with a temperature controlled autosampler, eluent degas module model 2465 electrochemical detector with 3 mm gold WE, and Hy-REF electrode. The column temperature was held at 29°C. The total run time was 100 min and mobile phase A consisted of 500 mM sodium acetate, mobile phase B consisted of 400mM sodium hydroxide, and mobile phase C consisted of 100% HPLC grade water.

### Statistical Analysis

Data was analyzed using SAS JMP version 10. Quantities of metabolites between conditions were compared using a student t-test. Metabolite levels for each treatment were compared to metabolite levels for control conditions, and significant differences were established at a p<0.05.

Initially, specific glucose consumption was calculated for each day by dividing the amount of glucose consumed per hour by the average number of cells at that time interval. It was noted that maximum specific glucose consumption always occurred in the first two days, during the exponential growth phase. Thus, to increase accuracy, a best-fit exponential curve was used for cell density. Final values for maximum specific glucose consumption were determined by equating the difference in glucose to a constant specific consumption rate multiplied by the integral of the total number of cells over the time period between when samples were taken. These samples occurred once daily.

In targeted transcriptomics analysis, relative gene expression values at large scale were compared for each gene to the amount of sialylation in each run. A linear fit was made for each gene to measure correlation between sialylation and gene expression. A p-value was found for each gene corresponding to the null hypothesis that no correlation existed between expression level and sialylation. A gene was considered significantly correlated at p<0.05.

## Results and Discussion

### Cell culture performance

**Fig 1:**
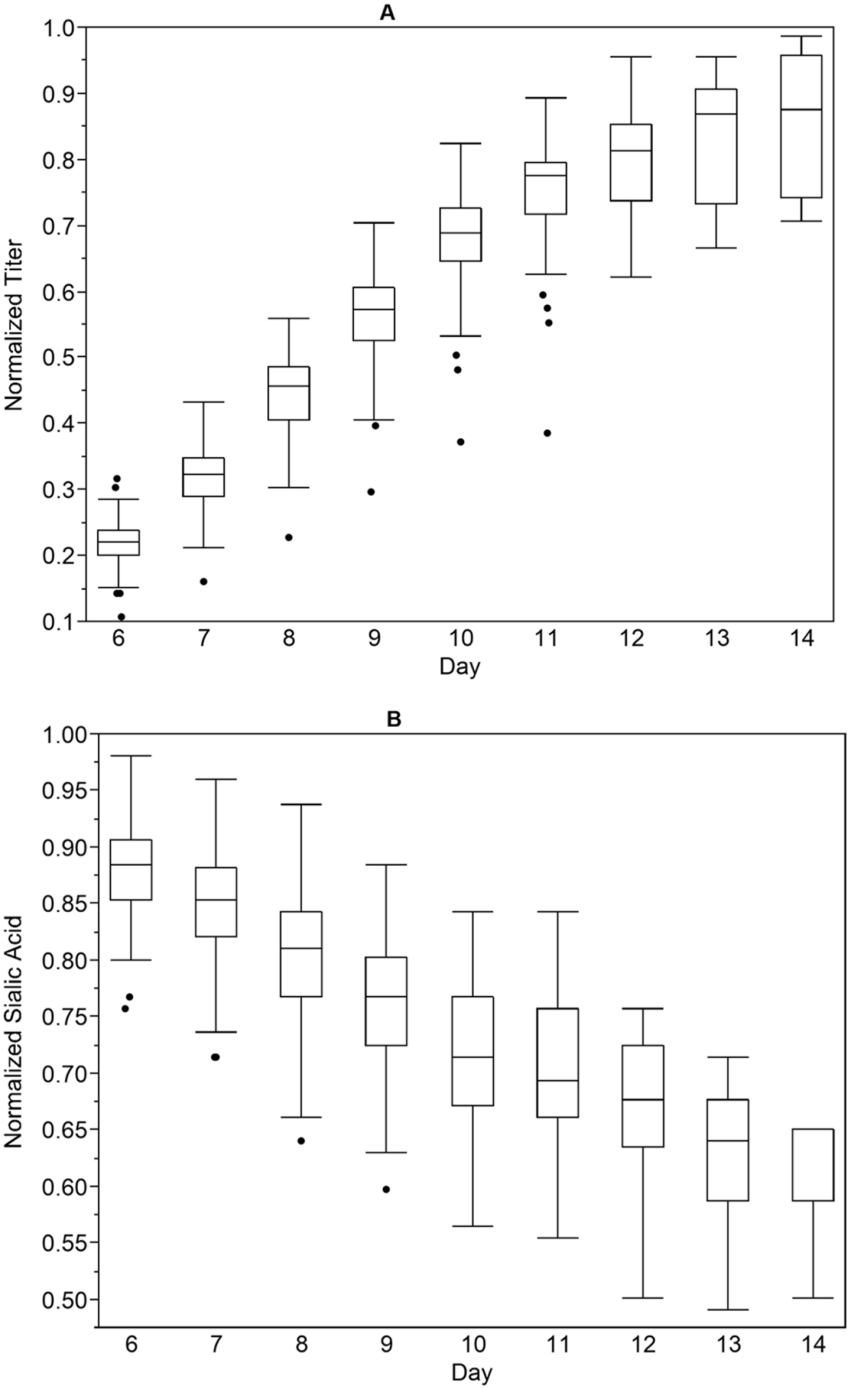
Large Scale Cell Culture Performance. Cell culture performance attributes titer (A) and NANA level (B) are shown over culture duration for 45 bioreactor runs at 50-L, 500-L, 900-L and 5000-L scale. Data was normalized to range from 0 to 1. Median is indicated by the horizontal line, interquartile range by the height of the box, and the full range by the vertical line. Outliers are shown as dots.

The performance of a fed-batch bioreactor process using a DG44 CHO cell line expressing a recombinant fusion protein was evaluated at pilot (50-L, 500-L, and 900-L) and production (5000-L) scales. Viable cell density (VCD), viability, gas (pCO_2_ and pO_2_), pH, osmolality, and metabolite (glucose, lactate, glutamine, glutamate and ammonia) levels were monitored daily and profiles were consistent run to run and across scales. Protein titer levels were measured daily starting on day 6. The sialylation level, as measured by the moles of N-acetylneuramic acid (NANA) per mole of protein, is an important quality attribute of the fusion protein and is monitored throughout the process starting on day 6. Thirty-six runs at pilot and nine runs at production scale were carried out using a single set of operating conditions. The performance and variation of these runs over culture duration is shown in Fig 1, with data normalized such that values range from zero to one. The titer (Fig 1A) increases linearly with time and is closely linked with VCD. There is minimal variation in titer levels between runs, and this variation is associated with differences in VCD. The sialylation profiles (Fig 1B) across the forty-five large scale runs varied independently of VCD, viability and titer. For the same culture duration, run to run variability of more than 2 NANA units was observed. The known assay variability for this measurement is less than 0.5 units, indicating that real variation for this attribute exists in the process. We were interested in identifying the source of this variability, and using that understanding to better control the process and achieve more robust and consistent sialylation levels.

### Untargeted transcriptomics at pilot and manufacturing scale

Cell culture profiles across pilot and manufacturing scale showed significant variation in sialylation profiles. Despite this variation in sialyation, no corresponding differences were observed in the monitored process attributes including cell growth, pH, dissolved gases or metabolite levels. In order to better understand the cause of this variation, untargeted metabolomics and transcriptomics were utilized as screening methods to better understand the process and identify potential clues relating to the observed variation. Gene expression in select pilot and manufacturing scale samples was quantified using global microarray. Cell pellets were prepared and treated as described in the Materials and Methods. A summary of the samples and their relative NANA levels is shown in Table 1. In order to identify expression of genes that correlated with NANA level, the six samples were given designations of ‘low,’ ‘medium,’ and ‘high’ sialic acid, based on what is typically observed in the process. An R-squared value was then calculated for each gene based on the linear fit between gene expression and NANA value. Averages were used for the medium and high sialic acid groups, which consisted of two and three samples respectively. The dataset was filtered for R-squared values greater than 0.9 and a base 2 log-fold change in gene expression greater than 1.5 or less than 0.67 between the low and high sialic acid groups. This analysis resulted in a filtered list of 34 potential markers and 16 annotated CHO genes. A summary of the genes and their known functions is presented in Table 2. The uneven distribution in sample size between groups is a result of our desire to use samples collected from a limited number of large scale runs representative of the manufacturing process.

**Table 1:** Process and NANA conditions of samples used for microarray.

Scale	Day	Relative NANA	NANA Group
900-L	10	-2.2	Low
5000-L	9	-1.0	Medium
5000-L	9	-0.7	Medium
500-L	10	-0.1	High
5000-L	9	-0.2	High
5000-L	9	0.0	High

Six samples from large scale runs ranging from 500-L to 5000-L and days 9 and 10 were used for global microarray analysis. Relative NANA levels spanned 2.2 units. Samples were grouped into low, medium and high based on normal NANA levels for this process.

**Table 2:** Global microarray targets correlated with NANA level.

Gene ID	R^2^	Log Fold Change (high to low)	Correlation with NANA	Description	Known Function(s)
BMP6	>0.999	1.69	Positive	Bone morphogenetic protein 6, secreted signaling protein	Induces growth of bone and cartilage, regulates iron homeostasis
ARHGEF15	>0.999	0.58	Negative	Rho GTPase	Signal transduction pathway regulator
ACTG1	0.998	0.62	Negative	Gamma-actin protein	Cell motility and cytoskeleton maintenance
SURF4	0.995	1.51	Positive	Surfeit locus protein 4, membrane protein	Facilitates transport between the ER and Golgi in mammalian cells
AKNA	0.994	1.56	Positive	AT-hook transcription factor	Upregulates CD40 and CD154
BTBD11	0.993	1.66	Positive	BDB/POZ domain, membrane protein	
ASNSD1	0.993	0.65	Negative	Asparagine synthetase domain	Glutamine metabolism, asparagine biosynthesis
MS4A14	0.991	0.63	Negative	Membrane spanning 4-domains A14, membrane protein	
PMP34	0.989	0.61	Negative	Peroxisomal membrane protein	ATP transporter of cofactors
ELP2	0.981	0.52	Negative	Elongator complex protein 2	Subunit of elongator complex (HAT), regulates response to environmental stress
TSPAN2	0.980	1.53	Positive	Tetraspanin-2, membrane protein	Associated with cell signaling, cell development, activation, growth and motility
TMOD1	0.978	0.60	Negative	Tropomodulin-1	Peroxidase, blocks elongation and depolymerization of actin filaments
HYDIN	0.977	0.52	Negative	HYDIN axonemal central pair apparatus protein	Ciliary motility
LHFP	0.974	0.65	Negative	Lipoma HMGIC fusion partner, membrane protein	
ZFN235	0.976	0.63	Negative	Zinc-finger nuclease 235	
ZFN420	0.972	1.55	Positive	Zinc-finger nuclease 420	

Statistical analysis was used to determine potential microarray targets correlated with NANA levels. The linear fit between gene expression and NANA level (R^2^), and log fold change between the high and low NANA groups were determined for each gene. Only genes with an R^2^ value great than 0.95 and log_2_ fold change greater than 1.5 or less than 0.67 were considered significant.

The expression of nine genes was found to be negatively correlated with NANA level, indicating higher gene expression in the low NANA conditions. PMP34 is a peroxisomal ATP transporter and is necessary for β-oxidation in mammalian peroxisomes [15]. Upregulation of PMP34 in low NANA conditions could indicate elevated peroxisomal activity. ASNSD1 is a domain of asparagine synthetase, which converts aspartate into asparagine using ATP and glutamine as precursors and generating AMP and glutamate as by-products. Human asparagine synthetase expression is known to increase in response to cellular stress, and is elevated in tumor cells under hypoxic conditions [16]. ELP2 is a subunit of the Elongator complex, a histone acetyltransferase that regulates gene expression and is well conserved across eukaryotes [17]. Previous work demonstrates that Elongator complex plays a critical role in environmental stress response, including oxidative stress [18,19]. TMOD1 reportedly possesses peroxisome activity and plays a role in antioxidant defense [20,21]. ARHGEF15 is a Rho GTPase involved in regulation of multiple signal transduction pathways. This family of proteins has been shown to be associated with oxidase regulation [22]. ACTG1, MS4A14, HYDIN, and LHFP were also negatively correlated with NANA level.

The expression of seven genes was found to be positively correlated with NANA level, indicating lower gene expression in the low NANA conditions. BMP6 is a secreted signaling and key regulatory protein in mammalian iron metabolism. A previous study in mice showed that BMP6 expression was decreased in response to oxidative stress [23]. BTBD11 encodes a protein-protein interaction domain found at the N-terminus of some zinc finger transcription factors [24]. In a study with human carcinoma cells treated with cadmium, which is thought to induce oxidative stress by depleting glutathione levels, BTBD11 gene expression was down regulated [25]. SURF4 is a membrane protein related to transport between the ER and Golgi in mammalian cells. AKNA, TSPAN2, ZFN235 and ZFN240 were also positively correlated with NANA level.

Several of these markers, including PMP34, ASNSD1, ELP2, TMOD1, ARHGEF15, BMP6 and BTBD11, have been demonstrated to be linked with oxidative stress and hypoxia. Previous studies have shown that in large scale bioreactors, insufficient oxygen mass transfer can exist, resulting in oxygen gradients [26]. Such oxygen gradients could result in intermittent hypoxia, increased reactive oxygen species (ROS) and the induction of cellular oxidative stress [27–30].

### Untargeted metabolomics at pilot and manufacturing scale

**Fig 2:**
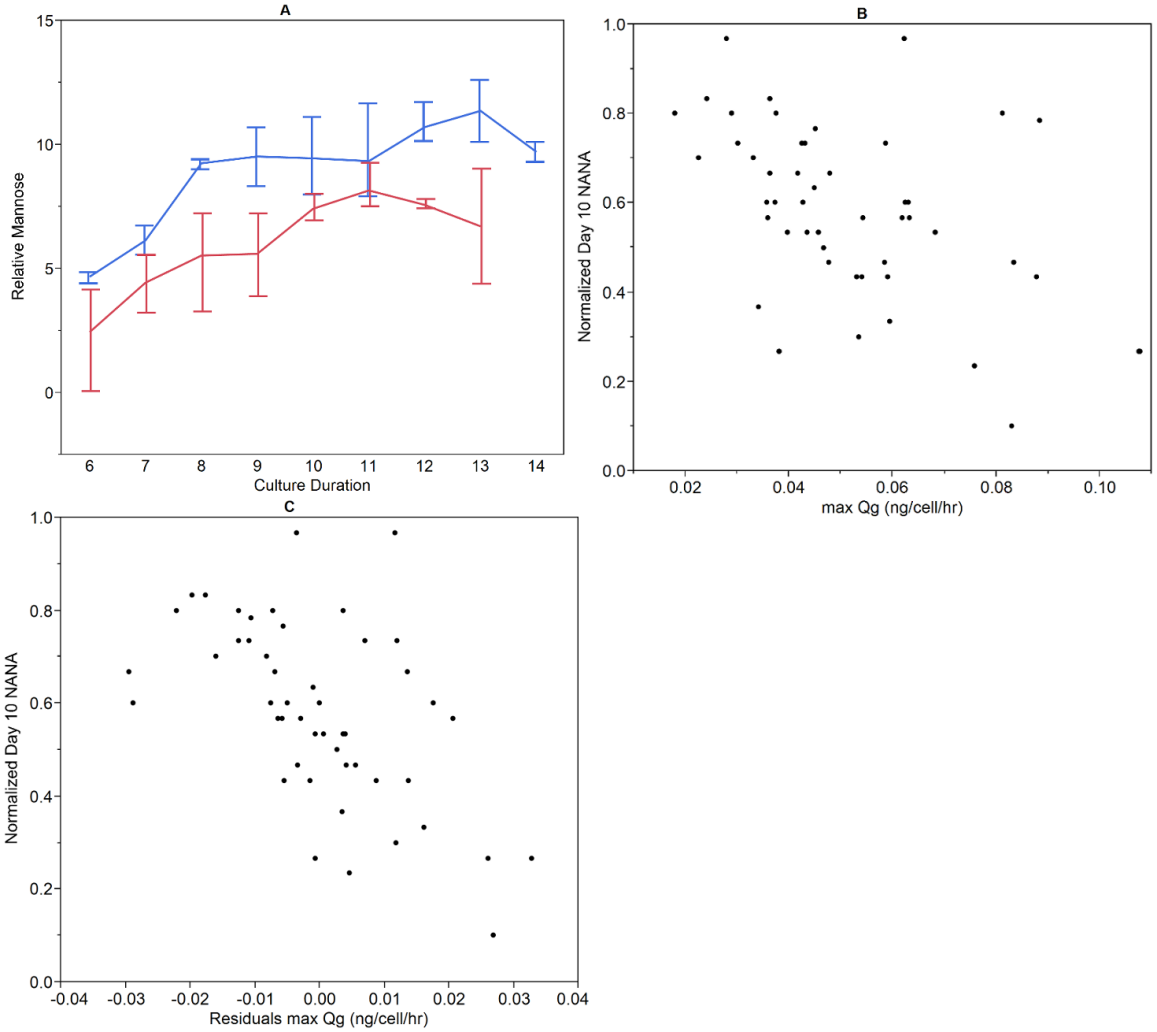
Links between glucose metabolism and NANA. Untargeted metabolomics & glucose consumption calculations were performed on samples ranging from 50-L to 5000-L scale. A. Relative mannose levels, a metabolite not provided in the cell culture media, were compared across runs with normal (red) and low (blue) NANA levels. Error bars represent the mannose range associated with each time point. B. Maximum specific glucose consumption was calculated for each run and found to be significantly inversely correlated to day 10 NANA (p<0.001). C. Residuals of maximum specific glucose consumption were calculated for each run and found to be significantly inversely correlated to day 10 NANA (p<0.0001).

The orthogonal approach of metabolomics was also applied to select samples. As described in the Materials and Methods section, metabolites in the cell culture supernatant were identified using NMR analysis. Samples were collected from two 5000-L runs, one 900-L run, and eight 50-L runs representing both normal (based on historical performance) and low sialylation profiles. A MANOVA repeated measures analysis in JMP was used to identify possible trends between metabolite levels and NANA. As shown in Fig 2A, extracellular mannose levels were found to increase over culture duration. Additionally, the relative mannose levels of low sialic acid runs is elevated compared to normal sialic acid runs. Peak mannose levels were quantified for select samples and found to be between 100 and 200 μM. In this fed-batch process, no mannose is present in the basal or feed media. Therefore, the accumulation of mannose in the supernatant is a by-product of cell metabolism.

Mannose is a key substrate in the N-glycosylation pathway and can be derived from fructose in a reversible reaction. The enzyme phosphomannose isomerase (PMI) converts fructose-6-phosphate to mannose-6-phosphate, which can then be directed into the N-glycosylation pathway [31]. Based on the shift in extracellular mannose levels between normal and low NANA levels, we hypothesized that glucose metabolism could be related to differences in sialylation observed at pilot and manufacturing scale. Glucose is provided to the cell line in both basal and feed medium. Specific glucose consumption levels were calculated as described in the Materials and Methods. As shown in Fig 2B, specific glucose consumption inversely correlates with day 10 NANA, (p<0.001) and runs with high specific glucose consumption had lower sialic acid, demonstrating a possible link between glucose metabolism and sialylation. The higher glucose consumption could be a result of a shift in cellular metabolism and relative increase in flux through glycolysis. While glycolysis generates less energy per glucose molecule, it requires less oxygen than oxidative phosphorylation and glucose is not limited in this process. No relationship between initial glucose concentration and day 10 NANA was observed (p>0.1), thereby indicating that glucose consumption is not simply a result of initial glucose concentration. Finally, the residuals of specific glucose consumption plotted against day 10 NANA are shown in Fig 2C. The residuals show an inverse correlation (p<0.0001). This indicates that the difference in specific glucose consumption is likely not causal in NANA level variation, but rather a second factor is related both to specific glucose consumption and to the observed variation in day 10 NANA at large scale. Taking into account the untargeted transcriptomics data that showed differences in gene expression associated with hypoxia, we hypothesized that intermittent hypoxia and the resulting cellular oxidative stress could be an additional factor influencing sialylation in the large scale process.

### Targeted transcriptomics at pilot and manufacturing scale

**Fig 3:**
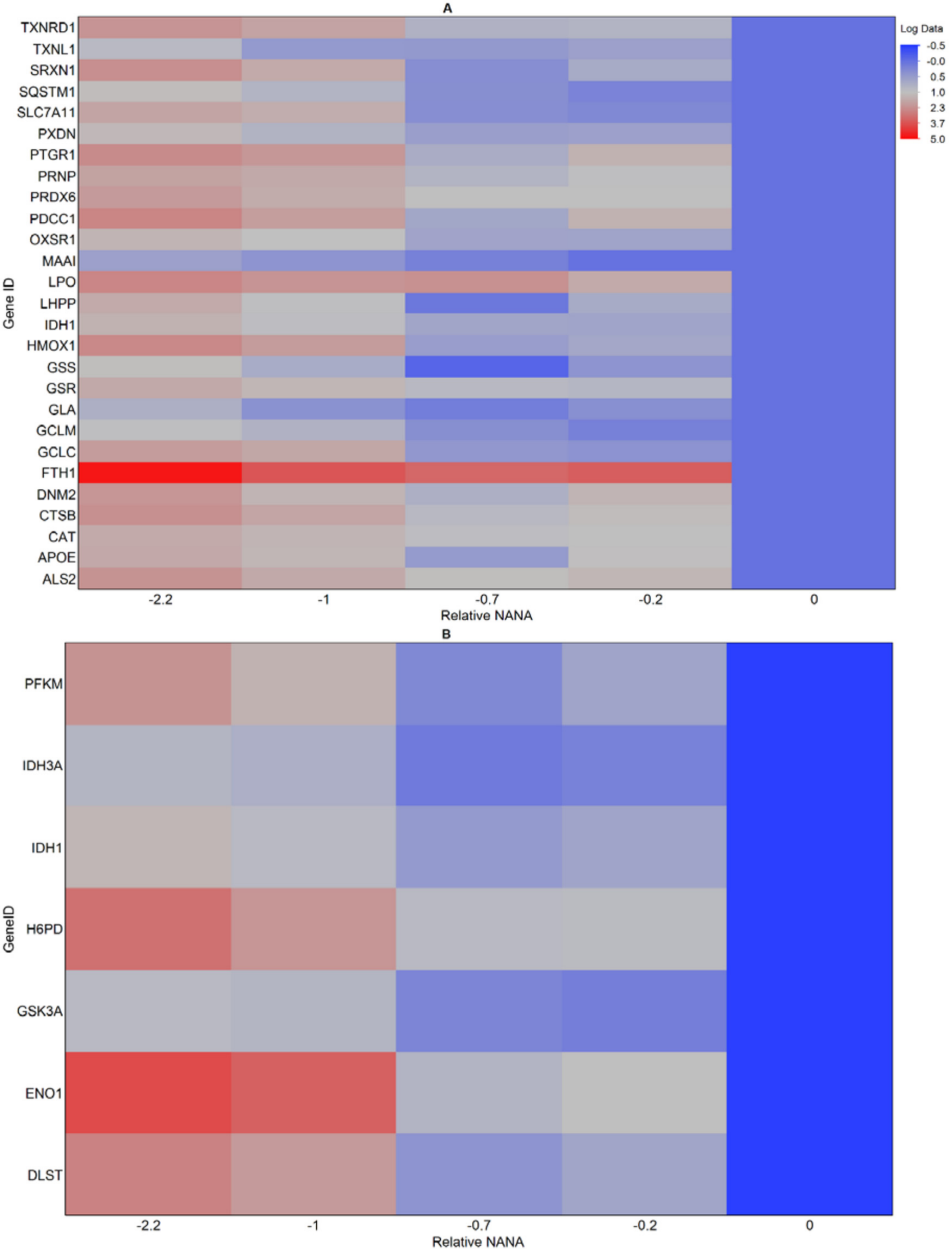
Targeted transcriptomics show upregulation of key oxidative stress and glucose metabolism genes at low NANA levels. PCR arrays were used for targeted transcriptomics of CHO oxidative stress pathway (A) and glucose metabolism (B). A P-value was found for each gene corresponding to the null hypothesis that no correlation existed between expression level and Relative NANA. Genes were considered significantly correlated at p<0.05. The log fold change in expression level between samples is indicated by color, where red indicates higher expression and blue indicates lower expression relative to the sample with the highest NANA level. The maximum change in expression is 5.5 log-fold.

Untargeted transcriptomics and metabolomics identified differential levels of metabolites and expressed genes across sialylation levels related to glucose metabolism and oxygen. In order to validate these findings, we performed targeted transcriptomics on five of the same pilot and manufacturing scale samples representing a NANA difference of 2.2 units. CHO-specific PCR arrays for glucose metabolism and oxidative stress were obtained from SABiosciences and prepared as described in the Materials and Methods. Each array measures expression of 84 pathway specific genes, and contains housekeeping genes and controls necessary for normalization and relative comparison. The correlation between each gene and sialylation level was determined by finding the best fit regression line between day 10 NANA level and gene expression at the same time point. Significance was determined when a p-value less than 0.05 was observed for the null hypothesis of the regression line having a slope equal to zero. Gene expression across samples was normalized to the sample with the highest NANA level. The twenty-seven oxidative stress genes with a statistically significant correlation to sialylation level are shown in Fig 3A. All genes are inversely correlated to sialic acid, indicating that when sialic acid is lower, expression of these oxidative stress markers is upregulated. Upregulation of these genes is consistent with increased oxidative stress, and the corresponding cellular response. Several genes associated with glutathione synthesis, a key antioxidant, are upregulated in the low versus high NANA conditions, including glutamate-cysteine ligase (both the catalytic -GCLC- and regulatory -GCLM- subunits), glutathione reductase (GSR), and glutathione synthetase (GSS). Several peroxidases, which catalyze the reduction of hydrogen peroxide, including catalase (CAT), lactoperoxidase (LPO), peroxidasin homolog (PXDN), and peroxiredoxin-6 (PRDX6), are upregulated at lower NANA levels. Additionally, sulfiredoxin-1, which reactivates peroxiredoxins, is upregulated at the lower NANA levels. Additional reducing enzymes including ferroxidase ferritin heavy chain 1 (FTH1) and thioredoxin reductase 1 (TXNRD1) and oxidative stress response 1, a regulator of downstream kinases in response to stress, are upregulated at lower NANA levels. Thioredoxin protein 1 (TXNL1) is also upregulated at the lower NANA levels. This response measured across large scale runs of varying NANA levels is strongly indicative of a link between sialylation and oxidative stress at the cellular level.

Gene expression of key glucose metabolism genes was also measured for the same large scale samples. The seven genes with a statistically significant correlation to sialylation level are shown in Fig 3B. All genes are inversely correlated to NANA level, indicating that when sialic acid is lower, expression of these glucose metabolic genes is upregulated. Two of the genes are associated with glycolysis and known to be upregulated in hypoxic conditions [32]: enolase 1 (ENO1) and 6-phosphofructokinase (PFK). PFK is a rate limiting step in glycolysis, thus upregulation of this enzyme indicates that in the low sialic acid condition, there is increased flux through glycolysis. This is consistent with the increased specific glucose consumption rates observed in low NANA conditions at large scale (Fig 2B). Interestingly, PFK expression may be linked to cellular oxidative stress. A study in mouse fibroblasts demonstrated *in vivo* interaction between PFK and nucleoredoxin (NRX), a thioredoxin protein known to be upregulated in response to oxidative stress [33]. Their findings indicate NRX, which is expressed in response to oxidative stress, has a regulatory role in PFK activity as well as the balance between glycolysis and the pentose phosphate pathway. Although NRX was not included in the glucose or oxidative stress arrays, differential expression of FTH1 and TXNL1 thioredoxins were detected.

Additionally, we observed upregulation of several genes associated with the citric acid cycle, including isocitrate dehydrogenase 3 (mitochondrial, IDH3A) and dihydrolipolyllysine-residue succinyltransferase (DLST), which is a subunit of the 2-oxoglutarate dehydrogenase complex (OGDC) in the mitochondria. OGDC, also known as α-ketoglutarate dehydrogenase (KGDH), is a key control point in the citric acid cycle. Researchers have shown that OGDC is sensitive to mitochondrial redox and could function as a redox sensor [34]. Studies in rats treated to generate a hypoxic condition *in vivo* showed OGDC is temporarily inhibited followed by up-regulation of gene expression [35]. This indicates that up-regulation of OGDC in the low NANA condition could be linked to oxidative stress. Cytosolic isocitrate dehydrogenase (IDH1), which is included in Fig 3A and 3B, converts isocitrate to 2-oxoglutarate and NADP to NADPH and localizes in the cytoplasm and peroxisomes. The NADPH generated by IDH1 is used in glutathione and peroxisomal reduction [36]. Hexose-6-phosphate dehydrogenase (H6PD) is also upregulated in the low NANA condition. H6PD catalyzes the first step of the pentose phosphate pathway (PPP) in the lumen of the endoplasmic reticulum, converting glucose-6-phosphate to 6-phosphogluconolactone. This conversion provides NADPH, a necessary cofactor for activity of luminal reductases [37]. A similar enzyme, Glucose-6-phosphate dehydrogenase (G6PD), has glucose specificity and catalyzes the first step in the oxidative phase of PPP in the cytosol and converts NADP+ to NADPH. In addition to its critical role in PPP regulation, G6PH plays an indispensable role in oxidative stress response in mammalian cells by generating the NADPH necessary for glutathione and peroxidase reactions to occur [38,39]. In this study, G6PD expression was inversely correlated with NANA level and close to the criteria specified for significance (p = 0.1).

### Impact of dissolved oxygen on laboratory scale cell culture

Pilot and manufacturing scale runs for an Fc-fusion protein process showed significant variation in sialylation profiles. Analysis of cell culture performance, metabolite levels, and gene expression at large scale provide some insight into cell metabolism and physiology, and indicate a link between sialylation level, glucose metabolism and oxidative stress. However, it is not practical to conduct experiments at either the pilot or manufacturing scale to directly test the role of oxygen on protein sialylation. In order to better understand the process, a 5-L bioreactor laboratory scale model was employed. The fed-batch process was executed as described in the Materials and Methods. As part of work performed separate from these studies, 5-L cell culture performance, titer and quality attributes were demonstrated to be consistent with the manufacturing scale process under control conditions using industry acceptable criteria [40].

**Fig 4:**
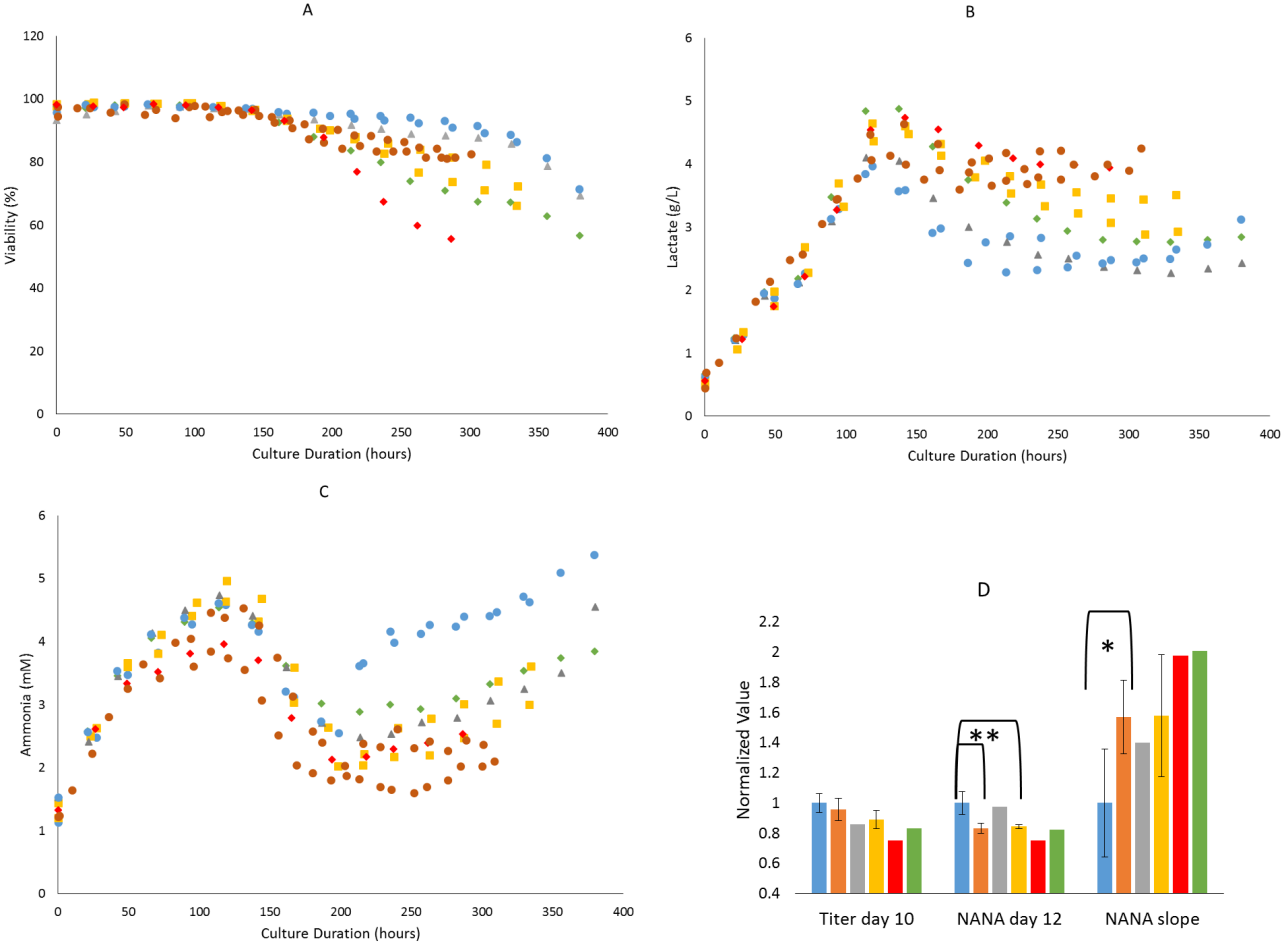
Reduced DO level impacts 5-L cell culture performance and sialylation. 5-L bioreactors were operated under control (50% DO, blue circle) and low DO (20% grey triangle, 15% yellow square, 10% red diamond, and 10% shifted to 20% DO on day 5 green diamond) conditions and compared to 5000-L operation (50% DO, orange circle). Viability (A), lactate (B) and ammonia (C) profiles were established for up to 14 days of bioreactor operation. D. Day 10 titer, day 12 NANA and NANA slope values were normalized to the 5-L control (50% DO) condition. Replicate bioreactors were used for control (n = 6), 5000-L (n = 8) and 15% DO (n = 4) conditions. Statistical differences were determined using a student t-test, * indicates p<0.05 and ** indicates p<0.01.

The process is typically carried out with a dissolved oxygen (DO) set point of 50% regardless of scale. Using the 5-L scale system, DO set points of 10%, 15%, 20%, and 10% shifted up to 20% on day 5 (10 to 20%) were tested in an effort to achieve oxygen levels closer to what cells may be effectively experiencing at larger scales. Several parameters in these low DO runs matched more accurately the behavior seen at large scale, offering further evidence for the periodically lower oxygen conditions experienced in large scale processes. Select cell culture profiles are shown in Fig 4. Fig 4A shows the culture viability, which is the same across all conditions until after peak VCD is reached. The 5-L control (50% DO set point) maintains a higher viability late in the run compared to the 5000-L scale. Reducing the DO results in reduced viability, with 20% having the highest viability and 10% DO having the lowest viability late in the run. The viability profile of the 15% DO condition very closely matches that of the 5000-L scale. Fig 4B and 4C show the lactate and ammonia profiles over culture duration. Data from 5000-L scale runs is included for comparison. Lactate accumulation at the beginning of the run is similar across all conditions. At the 5000-L scale, peak lactate levels are greater than 4 g/L, after which lactate levels plateau for the remaining culture duration. In contrast, peak lactate levels in the 5-L scale are less than 4 g/L and lactate is consumed late in the run. Reducing the DO results in increased peak lactate levels and reduced lactate consumption late in the run. The lactate profile of the 10% DO condition most closely resembles that of the 5000-L condition. Ammonia accumulation at the beginning of the run is similar across all conditions. At the 5000-L scale, peak ammonia levels are typically below 4 mM, after which ammonium levels decrease to below 3 mM for the remainder of the culture duration. In contrast, 5-L ammonium levels peak above 4 mM, after which ammonium levels drop below 3 mM before increasing above 4 mM late in the run. Reducing the DO results in reduced peak ammonium levels throughout the run. The ammonium profile of the 10% DO condition most closely resembles that of the 5000-L condition. These experimental results indicate that by changing DO set point, cell metabolism as indicated by viability, lactate, and ammonium profiles can be influenced. Reducing the DO level corresponds to decreased viability, increased lactate levels, and reduced ammonium levels, which is consistent with increased glycolysis and reduced amino acid catabolism.

Fig 4D shows the relative titer for the different conditions on day 10. Titer between the 5-L and 5000-L scales are equivalent (p = 0.12). The 20% and 15% DO (p = 0.12) condition resulted in no significant change to titer compared to both the 5-L and 5000-L conditions. The 10% DO and 10 to 20% DO conditions both resulted in reduced titer. This is likely because of the reduced viability and cell number late in the culture duration in the lower DO set point conditions. Fig 4D also shows the relative NANA levels on day 12 and the relative NANA slopes for the different conditions. NANA levels were observed to be on average 17% lower in the 5000-L scale compared to the 50% DO 5-L scale (p = 0.002). By reducing DO set point, NANA level is reduced in the 5-L model. Only a small difference (less than 3%) was observed in the 20% DO condition. The 15% DO condition resulted in a 15% decrease in NANA level compared to the 50% DO 5-L model (p = 0.003). There is no statistical difference between 5000-L scale and 15% DO at 5-L scale (p = 0.10). The 10% DO and 10 to 20% DO conditions further reduced the NANA level by 25% and 18% respectively. Finally, we looked at NANA slope over culture duration. Because NANA level is decreasing over the culture duration, NANA slope is a negative value. An increased normalized slope indicates NANA level is decreasing at a faster rate. Normalized NANA slope is 58% higher in the 5000-L scale compared to 5-L scale (p = 0.02). Reducing DO set point increases normalized NANA slope. The 20%, 15%, 10% and 10 to 20% DO conditions increased normalized NANA slope by 40%, 58%, 97% and 100% respectively. These results indicate that a reduced DO set point directly impacts NANA level and NANA slope. Furthermore, we identified a DO set point of 15% where NANA level and slope was impacted and more similar to 5000-L scale, but process titer was not changed. This indicates DO could be used to control NANA level independently of process yield.

### Targeted transcriptomics at laboratory scale

**Fig 5:**
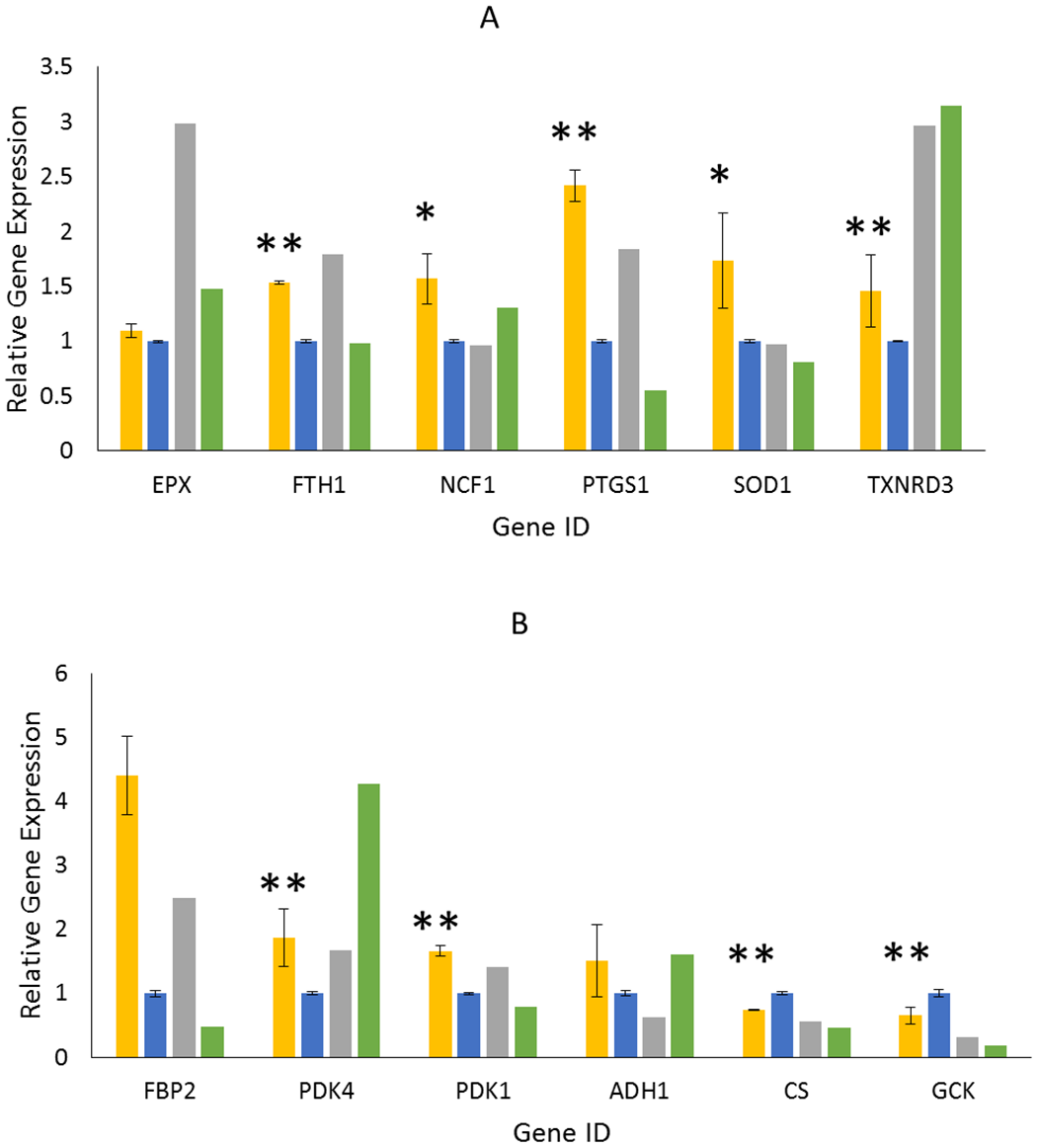
Reduced DO level impacts 5-L cell culture gene expression. 5-L bioreactors were operated under control (50% DO, blue) and low DO (20% gray, 15% yellow, and 10% shifted to 20% DO on day 5 green) conditions. Gene expression, relative to the control (50% DO) is shown for oxidative stress (A) and glucose metabolism (B) markers. Replicate bioreactors were used for control (n = 3), and 15% DO (n = 2) conditions. Statistical differences were determined using a student t-test, * indicates p<0.05 and ** indicates p<0.01.

The cell culture data shows DO has a direct impact on sialylation level in the laboratory scale model. In order to link this laboratory scale data with the manufacturing scale process, we used both oxidative stress and glucose metabolism PCR arrays to measure gene expression. Relative expression of oxidative stress markers across the DO set points at 5-L scale is shown in Fig 5A, with n = 3 replicates for the 50% DO, n = 2 replicates for the 15% DO, and n = 1 for 20% DO and 10 to 20% DO treatment. Despite the phenotypic changes as evident in the cell culture data, gene expression was less impacted by the low DO treatment at 5-L compared to the 5000-L scale. In particular, no significant difference was observed in CAT, GSS, GSR or PRDX genes. This indicates that reduced DO set point in the 5-L scale may not fully recreate the hypoxic condition experienced at 5000-L scale. Despite differences in gene expression, we did observe upregulation of select genes that indicate some oxidative stress response in the conditions with reduced DO set point. Eosinophil peroxidase (EPX), a heme peroxidase, was significantly upregulated in the 20% DO and 10 to 20% DO conditions, and slightly upregulated in the 15% DO condition (p = 0.06) compared to control. FTH1 was significantly upregulated in both the 20% and 15% DO conditions (p<0.001), and is consistent with observations at 5000-L scale (Fig 3A). Neutrophil cytosol factor 1 (NCF1), which encodes a subunit of an NADPH oxidase, was significantly upregulated in 15% (p = 0.02) and 10 to 20% DO conditions. A previous study with rat cells, which were treated with cerium oxide to induce oxidative stress and antioxidant defense, similarly observed upregulation of NCF1 [41]. Prostaglandin-endoperoxide synthase 1 (PTGS1) was significantly upregulated in the 20% and 15% DO conditions (p<0.001). PTGS1 was also upregulated at 5000-L scale in the low NANA conditions, and close to meeting the criteria for significance. Superoxide dismutase 1 (SOD1), which is an established marker for oxidative stress, was significantly upregulated in the 15% condition (p = 0.05) and elevated in the 20% and 10 to 20% DO conditions as well. Finally, thioredoxin reductase 3 (TXNRD3) was upregulated in all low DO conditions compared to control.

Relative expression of glucose metabolism genes across the two scales and DO set points is shown in Fig 5B. Again, we observed that the majority of the glucose metabolism markers were not significantly impacted by the low DO set point. Further, we did not see changes in gene expression for any of the seven markers linked with NANA level (Fig 3B). Despite differences in gene expression at 5-L and 5000-L scales, we did observe upregulation of four genes and downregulation of two genes that indicate a shift in glucose metabolism that is consistent with an oxidative stress response. Alcohol dehydrogenase 1 (ADH1) expression, which is known to be induced under hypoxic conditions [42], was upregulated in both the 15% and 10 to 20% DO conditions. Fructose-1,6-bisphosphatase (FBP2) was upregulated in both 15% (p = 0.002) and 20% DO conditions. FBP2 expression is associated with increased cellular glutathione levels, and has been shown to increase in response to oxidative stress in multiple mammalian cell types [43,44]. Pyruvate dehydrogenase kinase (PDK1 and PDK4), which catalyzes oxidative decarboxylation of pyruvate, was upregulated in all low DO conditions (p = 0.02 for PDK4, p<0.001 for PDK1, 15% DO). Previous studies have shown that the HIF-1 protein, critical for hypoxic adaptation, activates PDK1. Furthermore, forced PDK1 expression rescues cells from hypoxic apoptosis [45]. Glucokinase (GCK), which phosphorylates glucose to form glucose-6-phosphate, is downregulated in all low DO conditions (p = 0.005, 15% DO). Although this result was not expected, previous work has demonstrated that GCK, as well as other genes associated with carbohydrate metabolism, can be repressed in response to oxidative stress [46]. Finally, we observed decreased citrate synthase (CS) expression in all low DO conditions compared to the control (p<0.001, 15% DO). CS activity is commonly used to measure the level of intact mitochondria, therefore decreased CS expression could indicate decreased mitochondria on a per cell basis in the low DO conditions.

### Metabolomics at laboratory scale

We were further interested in determining the intracellular metabolomic profiles of laboratory scale runs. Cell culture extracts were saved from 50% DO and 15% DO conditions, and prepared and analyzed as described in the Materials and Methods. One hundred thirty-three metabolites were detected and quantified using in house standards. Duplicate conditions at a single time point corresponding to peak VCD was used. Key metabolites and fold change relative to the 50% DO condition are shown in Table 3. Metabolites found significant (p-value ≤ 0.05) and nearly significant (p-value ≤ 0.10) according to a student t-test are included. Two nucleotide sugars, AMP and CDP, were found to be reduced in the 15% DO condition. The levels of these nucleotide sugar building blocks are known to be influenced by glucose metabolism [4]. NADP, taurine, and glutamyl-isoleucine, which are related to glutathione metabolism, were also reduced in the 15% DO condition. Reduced intracellular NADP levels have previously been observed in hypoxic cells [47]. N-acetylgalactosamine (GalNAc) and N-acetylglucosamine (GlcNAc) were 34% and 35% lower respectively in the 15% DO condition. Both acetylated sugars are key building blocks for N-glycans and are generated from glucose via the hexosamine biosynthetic pathway. GlcNAc is a pre-cursor for the charged form of sialic acid, CMP-NANA, which is transported into the Golgi for terminal N-glycan sialylation. Reduced intracellular levels of UDP-GlcNAc, which is generated from GlcNAc, and low sialylation levels have been observed under hypoxic conditions [4,32]. Intracellular NANA was 16% lower in the 15% DO condition compared to 50% DO condition. We were also interested in the relative levels of mannose, which we previously observed to be elevated in the supernatant of lower sialylation large scale samples. Intracellular mannose levels were 2.1 fold higher in the 15% DO condition. Extracellular mannose was also measured over 5 time points and found to be between 1.56 and 1.97 fold higher in the 15% DO condition. These observations at laboratory scale are consistent with mannose measurements from pilot and manufacturing scale. Furthermore, previous studies have shown that mannose-6-phosphate, a mannose pre-cursor generated from fructose-6-phosphate, is elevated in cells under hypoxic conditions [47,48].

**Table 3:** Intracellular metabolites impacted by oxygen treatments.

Metabolite	Fold-change, relative to 50% DO	p-value
14–0 LPC myristoyl-lyso-PC	1.59	0.02
16–1 LPC palmitoleoyl-lyso-PC	1.52	0.01
Acetyl-Phenylalanine	0.77	0.09
AMP	0.48	0.09
CDP	0.33	0.04
glutamyl-isoleucine	0.74	0.06
isobutyryl carnitine (C4)	0.46	0.04
isovaleryl carnitine (C5)	0.28	0.05
N-acetylgalactosamine	0.66	0.06
N-acetylglucosamine	0.65	0.03
NADP	0.67	0.06
phosphocholine	0.68	0.03
taurine	0.52	0.07

### Impact of dissolved oxygen on N-glycan structures

**Fig 6:**
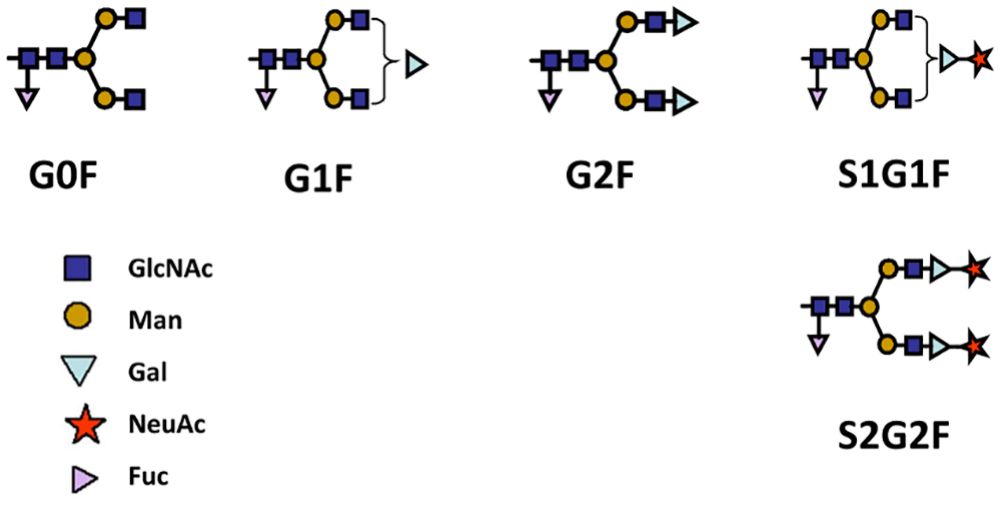
N-Glycan Structures. The N-glycan assay method quantifies the percent distribution of N-glycan species. Five key structures were quantified in samples generated from 5-L bioreactors were operated under normal (50%) and low (15%) DO conditions. G0F, G1F and G2F are unsialylated, S1G1F is monosialylated, and S2G2F is disialylated.

We were interested in understanding how oxygen treatments impacted the distribution of key N-glycan species in our product. Samples from days 10 and 14 were treated for N-glycan analysis as described in the Materials and Methods. The peaks corresponding to G0F, G1F, G2F, S1G1F and S2G2F were quantified for each sample in order to determine the percentage of each N-glycan species relative to total N-glycans. G0F is a common N-glycan structure with two branches each terminating in an N-acetylglucosamine (GlcNAc) residue. The G1F structure is formed by the addition of a galactose residue to one of the G0F branches, and the G2F structure is formed when galactose residues are added to both branches. NANA residues can be added to either branch once galactosylation has occurred. Terminal sialylation of the G1F structure forms S1G1F. Terminal sialylation of both branches in the G2F structure forms S2G2F. The chemical structure of these glycan species is shown in Fig 6. These values are presented in Table 4 and are reported relative to the 5-L control condition such that a positive value indicates a higher percentage of that species and a negative value indicates a lower percentage of that species in the 50% DO compared to 15% DO conditions.

**Table 4:** N-Glycan distribution for 5-L treatments.

Treatment	Day	G0F (Δ%)	G1F (Δ%)	G2F (Δ%)	S1G1F (Δ%)	S2G2F (Δ%)
20% DO	10	0.3	2.5	2.1	3.3	7.4
15% DO	10	-0.4 ± 0.2	1.4 ± 0.7	1.51 ± 0.3	0.6 ± 0.3	-1.7 ± 0.2
10 to 20% DO	10	0.6	0.8	1.5	0.6	0.48
20% DO	14	-1.4	0.4	1.2	-0.4	-2.1
15% DO	14	0.1	2.9	2.4	-3.5	-6.2
10 to 20% DO	14	-0.6	0.6	1.9	-0.1	-4.3

G0F, G1F, G2F, S1G1F and S2G2F species were quantified as a percentage of total N-glycans for days 10 and 14. The difference for each species between the treatment and the control group was determined and is reported as the change in percentage (Δ%). Positive values indicate a higher percentage of that species in the treatment compared to control, and negative values indicates a lower percentage of that species in treatment compared to control.

A previous study with mouse cells examined the impact of reduced DO levels on N-linked glycosylation of an IgG1 mAb. They observed that galactosylation was reduced under low DO conditions [13]. Because galactose must be added to N-glycan structures in order for sialylation to occur, reduced galactosylation could cause reduced sialylation. If that were the case in these studies, we would expect to see elevated levels of G0F and reduced levels of G1F and G2F in our 15% DO condition compared to the 50% DO condition. However, our results shows that G1F and G2F levels were higher at low DO conditions compared to 50% DO on both day 10 and day 14. Although the increased levels of G1F and G2F did not exceed 3%, this indicates that galactosylation is not limiting in this system. Thus, the reduced sialylation associated with low DO conditions is likely a result of N-glycan sialylation being limited, rather than galactosylation being limiting. On day 10, the S1G1F levels are comparable or slightly higher for the low DO treatments relative to the control. However, S2G2F levels were reduced in both the 15% DO and 20% DO conditions. On day 14, the S1G1F and S2G2F levels are reduced in all three low DO conditions. This indicates that over time, less G1F and G2F structures are terminally sialylated, resulting in lower measured NANA values for the entire protein pool.

**Fig 7:**
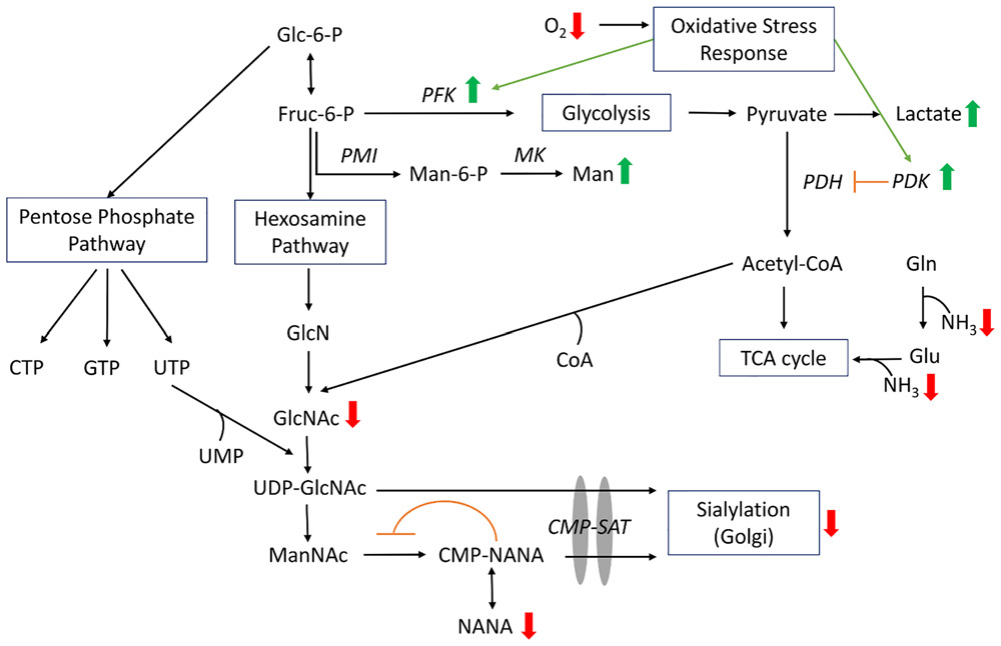
Proposed biological mechanism for manufacturing and low oxygen laboratory scale bioreactors. Reduced oxygen levels trigger an oxidative stress response and shift in glucose metabolism, resulting in upregulation of glycolysis and mannose synthesis, and downregulation of the hexosamine pathway and acetyl-CoA formation. This metabolic shift results in reduced GlcNAc levels, as well as levels of metabolites formed from GlcNAc including UDP-GlcNAc and CMP-NANA, triggering a reduction in terminal sialylation of N-glycans. Metabolite or gene expression levels directly measures as increased (green arrows) or decreased (red arrows) are shown. Abbreviations are as follows: CMP, cytidine monophosphate; CMP-SAT, CMP sialic acid transporter; CoA, coenzyme A; CTP, cytidine triphosphate; Frc-6-P, fructose-6-phosphate; Glc-6-P, glucose-6-phosphate; GlcN, glucosamine; GlcNAc, N-acetyl glucosamine; Gln, glutamine; Glu, glutamate; GTP, guanosine triphosphate; Man, mannose; Man-6-P, mannose-6-phosphate; ManNAc, N-acetyl mannosamine; MK, mannosekinase; NANA, N-acetylneuraminic acid; NH_3_, ammonia; PDH, pyruvate dehydrogenase; PDK, pyruvate dehydrogenase kinase; PFK, phosphofructokinase; PMI, mannose phosphate isomerase; UDP, uridine diphosphate; UMP, uridine monophosphate; UTP, uridine triphosphate.

Using the 5-L model system, we saw very clearly that DO set point impacted cell culture performance and protein glycosylation. Furthermore, by modifying the DO set point, we were able to achieve profiles more consistent with 5000-L manufacturing scale. Using metabolomics and transcriptomics, we measured changes in metabolites and gene expression for key markers associated with oxidative stress and glucose metabolism. Finally, analytical analysis of the N-glycan structures eliminated galactosylation as a potential root cause for the reduced sialylation under low DO conditions. These observations generated using a laboratory scale model are consistent with the hypothesis that the 5000-L phenotype is an oxygen limited, oxidative stress environment. Oxidative stress is likely a result of mixing limitations at large scale and the presence of oxygen dead-zones in the large scale bioreactor [26]. Furthermore, oxidative stress based gene regulation is known to be integrated in the physiological regulation of carbon metabolism [46]. Here, we observe a clear link between oxidative stress, glucose metabolism and protein sialylation. Based on the data generated in these studies and previously published studies, we have developed a proposed biological mechanism linking oxygen, glucose metabolism and protein sialylation, as shown in Fig 7. Reduced oxygen levels resulting in intermittent hypoxic conditions in the manufacturing scale bioreactor induce a cellular oxidative stress response, as demonstrated by transcriptomics data. This stress response results in changes to glucose metabolism including increased flux of glucose through glycolysis and mannose metabolism [47,48], and reduced flux through the pentose phosphate and hexosamine pathways. This is consistent with the observed increased lactate and reduced ammonia levels in low DO laboratory scale experiments. Oxidative stress also triggers upregulation of rate limiting glycolysis enzymes PFK and ENO1 [32], and PDK, which downregulates PDH, resulting in reduced formation of acetyl-CoA from pyruvate [32,47]. Acetyl-CoA is a precursor for GlcNAc formation, thus the reduced intracellular acetyl-CoA levels result in a lower intracellular GlcNAc pool, as measured by metabolomics. Reduced GlcNAc levels likely cause reduced levels of all subsequent metabolites in the hexosamine pathway, including NANA. The formation of CMP-NANA from ManNAc, which occurs in the cytosol, is feedback inhibited by CMP-NANA. This would further reduce the intracellular pool of NANA sialic acid used for N-glycan sialylation, which is consistent with metabolomics data.

## Conclusions

This study examines a CHO bioprocess at 5000-L scale, where the product is a sialylated Fc-fusion protein. Variation in sialylation was observed to be more than four times higher than known assay variability across pilot and manufacturing scale runs, indicating variability in the process. Untargeted metabolomics and transcriptomics methods were applied to select pilot and manufacturing scale samples, which showed a shift in glucose metabolism and increased oxidative stress response at lower sialylation levels. Using a 5-L scale model, we were able to reproduce the phenotypic profiles observed at manufacturing scale, including lower sialylation levels, by reducing the dissolved oxygen set point. Targeted transcriptomics and metabolomics confirmed that we were able to alter metabolism and gene expression similarly to the manufacturing scale. Finally, we were able to develop a proposed mechanism explaining the phenotypic variation at manufacturing scale. Oxidative stress can be attributed to mixing limitations at large scale and the presence of oxygen dead-zones in the large scale bioreactor [26]. This induces upregulation of glycolysis and mannose biosynthesis, and downregulation of hexosamine biosynthesis and acetyl-CoA formation. The lower flux through the hexosamine pathway and reduced intracellular pools of acetyl-CoA cause reduced formation of GlcNAc and NANA, resulting in reduced N-glycan terminal sialylation. This study is an example of how ‘Omics tools can be applied to industrial bioprocessing to solve complex problems [11]. In this case, process, analytical, metabolomic and transcriptomic data was taken together to develop a systems level understanding of the process at the manufacturing scale.

The outcome of this study is a biological understanding of the previously unknown variation in a CHO bioprocess resulting in inconsistent sialylation. This biological understanding can now be used to identify key biomarkers for improved process monitoring and to develop strategies to reduce sialylation variation. The metabolomic and transcriptomic data sets can be leveraged for biomarkers. Simultaneous upregulation of PDK, PFK and oxidative stress markers such as SOD, CAT, GSS, GSR or FTH1 could collectively be a useful biomarker and could be easily monitored at any production scale using RT-PCR. In this system, extracellular mannose concentration would be a useful biomarker for monitoring process performance. Intra and extracellular levels of mannose were found to be inversely correlated with sialic acid levels. Direct measurement of sialic acid cannot be accurately performed in real-time, however mannose could be measured as an indirect indicator of sialylation level. Off-line monitoring can be achieved using HPLC [49] or enzymatic [50] methods. Alternatively, in-line monitoring could be achieved using a spectroscopic method, such as Raman spectroscopy. Although there has been limited application of these technologies at manufacturing scale to date, there have been significant advancements and growing interest from industry for such monitoring tools [51].

This study can also be leveraged to develop strategies and improvements that reduce run to run sialylation differences. From a process perspective, changes can be made to increase dissolved oxygen level, mixing and agitation, and bioreactor type and configuration. From a cell line development perspective, genetic modifications could be made to reduce the impact of a low oxygen environment. Specifically, gene overexpression could be used to increase flux through the hexosamine pathway and to increase PDH levels and subsequently acetyl-CoA intracellular pools. The shift towards glycolysis could be mitigated and transcription levels of PDK and PFK could be reduced by replacing their endogenous promoters with a weak, synthetic promoter. Finally, the outcome of both process and genetic modifications could be monitored in real time using the proposed biomarkers and 5-L laboratory scale model discussed here.
